# Validation of the food compass score through 24 h recalls and measurement of erythrocyte fatty acids in a mediterranean population

**DOI:** 10.1007/s00394-026-03912-0

**Published:** 2026-02-25

**Authors:** P. Detopoulou, M. Yannakoulia, E. Fragopoulou, N. Kalogeropoulos, T. Nomikos, S. Antonopoulou

**Affiliations:** Department of Nutrition and Dietetics, Harokopio University, 70 El. Venizelou Street, 17671 Athens, Greece

**Keywords:** Food compass score, Erythrocyte fatty acids, Validation, Nutrient profiling system

## Abstract

**Purpose:**

The Food Compass Score (FCS) is a newly introduced Nutrient Profiling System for assessing overall diet quality. Its validation against dietary intake and objective biomarkers is limited. This study aimed to validate the FCS using 24-hour recalls, erythrocyte fatty acids, a long-term biomarker of fatty acids intake, and MedDietScore as a healthiness index.

**Methods:**

Apparently healthy subjects were recruited. Two 24 h-recalls and a food-frequency questionnaire were administered. Erythrocyte fatty acids were determined by gas chromatography. Energy adjusted Spearman correlations were performed. In addition, simple and age/sex/BMI adjusted linear regression models were applied with FCS as dependent and energy-adjusted nutrients or biomarkers as independent variables.

**Results:**

One hundred and six subjects (48 men, 44 ± 13 years; 58 women, 44 ± 14 years) participated. The mean FCS of the study participants was 55.9 ± 9.5. In fully adjusted models the FCS was positively associated with % monounsaturated fatty acids (% of total energy intake) (B = 0.401, 95% CI:0.170–0.632, *p* = 0.001), % fat (% of total energy intake) (B = 0.230, 95% CI:0.036–0.425, *p* = 0.02), vitamin C (B = 0.022, 95% CI:0.005–0.038, *p* = 0.01), ɑ-tocopherol (B = 0.427, 95% CI:0.0070–0.785, *p* = 0.02), vitamin K (B = 5.225, 95% CI:1.670–8.840, *p* = 0.005), potassium intakes (B = 0.002, 95% CI:0.0008–0.004, *p* = 0.04), and the MedDietScore (B = 0.805, 95% CI:0.473–1.137, *p* < 0.001). It was also negatively associated with % carbohydrate intake (% of total energy intake) (B = − 0.209, 95% CI: − 0.391 to − 0.027, *p* = 0.025). In addition, the FCS was associated with unsaturated/ SFA ratio, dietary fiber/carbohydrate ratio, and potassium/sodium ratio after adjustment for age, sex, BMI and energy intake. The FCS was positively associated with the % erythrocyte EPA (B = 13.814, 95% CI:5.632–21.996, *p* = 0.001), DHA (B = 1.769, 95% CI:0.332–3.205, *p* = 0.01), omega-3 fatty acids (B = 1.380, 95% CI:0.333–2.428, *p* = 0.01) and the omega-3 index (B = 1.581, 95% CI: 0.358–2.805, *p* = 0.01).

**Conclusion:**

The correlations of the FCS with the MedDietScore, nutrients and erythrocyte fatty acids investigated suggest that FCS is a valid index encouraging its use for clinical and consumer guidance.

**Supplementary Information:**

The online version contains supplementary material available at 10.1007/s00394-026-03912-0.

## Introduction

Several Nutrient Profiling Systems (NPS) have been developed to assist in formulating policy actions, new food product development and help consumers with food choices [1]. In addition, data point to a relation of NPS to chronic diseases and mortality [2–5]. The recently designed and updated Food compass score (FCS) is a promising NPS addressing previously reported issues regarding NPS [6, 7]. It incorporates 54 food characteristics in the following areas: nutrient ratios, food ingredients, additives, degree of food processing, lipid, fiber, protein, micronutrients and phytochemicals content, enabling a “holistic” evaluation of each food [6]. The presence of phytonutrients, vitamin D, choline, omega-3 and medium-chain fatty acids is positively scored, while additives and high degree of processing based on the NOVA score are negatively scored [6]. It is also noted that several ratios are included rather than absolute nutrient values, i.e. unsaturated to saturated fat ratio, carbohydrate to fiber ratio and potassium to sodium ratio, which possibly better reflect diet quality [6]. Our group has also reported positive correlations between FCS and juices, high fat dairy, vegetables, legumes, fruits and olive oil, and negative ones with sodas, alcoholic drinks, red meat, refined grains, sweets, fats other than olive oil, fast foods and coffee [8]. Moreover, the FCS has been positively associated with other quality indices, such as the Mediterranean Diet Score and the Health Star Rating [8].

Self-report methods, such as 24-h recalls, are an extensively used method for estimating daily energy, macronutrient, and micronutrient intakes; however they are susceptible to self-reporting bias [9, 10]. Conversely, nutritional biomarkers are gaining popularity and are considered as gold standard for validating the intake of specific nutrients. For example, erythrocyte fatty acids provide an excellent long-term biomarker of habitual fatty acid consumption. Indeed, erythrocytes contain saturated fatty acids (SFA), monounsaturated (MUFA), and polyunsaturated fatty acids (PUFA) and are considered a long term nutritional biomarker, since they reflect dietary fatty acid intake for a time interval of about 120 days [11]. Much attention, in addition, has been drawn to the content of erythrocytes in the omega-3 fatty acids eicosapentaenoic (EPA) and docosahexaenoic (DHA) as well as their sum as a percent of total fatty acids (% EPA + DHA), also known as the Omega-3 Index [12].

A recently published meta-analysis including studies up to 2022, has compared the “performance” of 9 NPSs against disease risk and relevant markers (criterion validity) [13]. However, the validation of existing NPS through dietary and nutritional biomarkers is mostly lacking, while data regarding the relationship of several diet quality scores to dietary or biochemical methods are scarce [9, 14]. Although the FCS has been inversely related to cardiovascular disease [15], dyslipidemia/obesity [16] and mortality [17], its convergent construct validity in terms of dietary factors has not been established. To our knowledge, there is no study addressing the correlation of FCS with nutrient intake and/or nutritional biomarkers.

Therefore, the aims of the present study were (i) to validate the FCS with reported total energy and nutrient intake with the use of 24 h recalls, (ii) to validate the FCS ability to capture long-term fatty acid consumption through its correlation with erythrocyte fatty acids, as a gold-standard biomarker for fatty acids intake, and to (iii) compare the FCS’s performance against the MedDietScore, a valid score reflecting dietary quality and healthiness.

## Methods

### Participants

The recruitment of volunteers was performed in the greater Athens area. The inclusion criteria were age > 18 years, and consent to participate in the study. The exclusion criteria were history of cardiovascular disease, history of chronic inflammatory conditions, acute respiratory problems, dental problems and renal/hepatic abnormalities, active cold or flu, use of food supplements, and the use of fish oils or medical treatment. The Bioethics Committee of Harokopio University approved the study protocol. The study was conducted in accordance with the Declaration of Helsinki (6th revision, 2008) of the World Medical Association. All participants gave their written informed consent. Blood was drawn in the morning after fasting for 9–12 h. Trained dietitians took anthropometric measurements and performed dietary assessment, as described below.

### Biochemical measurements

Fasting serum glucose, triacylglycerols, total-cholesterol and HDL-cholesterol were determined (ACE analyzer, Schiapparelli Biosystems, Inc, New Jersey, USA) using reagents from Alfa Wassermann (Woerden, The Netherlands). LDL-cholesterol was calculated with the Friedewald Formula. Moreover, serum insulin levels were determined with a commercially available ELISA kit (Invitrogen, Paisley, UK).

### Erythrocytes fatty acids determination

Erythrocyte fatty acids, particularly EPA and DHA, were selected as biomarkers because they reliably reflect long-term dietary intake of omega-3 fatty acids [11]. The protocol for erythrocytes isolation, extraction and methylation of fatty acids has been previously described [18]. The obtained fatty acids methyl esters (FAME) were extracted with hexane containing internal standard and butylated hydroxytoluene was added to prevent lipid peroxidation. Gas chromatography of FAME was used to determine fatty acids in erythrocytes (Agilent HP-6890 gas chromatograph equipped with flame ionization detector, Avondale, PA, USA). Separation of FAME was achieved on a BPX70 capillary column coated with cyanopropyl silicone (SGE, Melbourne, Australia). Helium was used as the carrier gas and injector and detector were held at 230 and 290° C, respectively. Peaks identification was accomplished with the use of a standard mixture of 37 FAME (Sigma L9405, St Louis, MO, USA). In the current work *trans* isomers were not determined. The omega-3 index was calculated as the sum of % content of erythrocytes in EPA and DHA [12].

### Anthropometry and body composition analysis

For the measurement of weight (in kg) and height (in cm) subjects wore light clothing and took their shoes off. Body mass index (BMI) was then calculated as the ratio of weight divided by height squared. Waist circumference was measured to the nearest 0.1 cm at the approximate midpoint between the last palpable rib and the top of the iliac crest.

Dual-energy-X-ray absorptiometry (DXA) was used for body composition analysis (Lunar Corporation, Model DPX1, Lunar Corp., Madison, WI)). The Lunar software (version 4.7e) was used for the analysis. Besides the standard analysis, a manually defined “region of interest” (ROI) was determined around the L1-L4 area representing abdominal fat [19].

### Dietary assessment

Two non-consecutive multiple-pass 24 h-recalls were collected and a semi-quantitative food frequency questionnaire (FFQ) with150 items was administered [20]. The 24 h-recalls were analyzed with the use of the Nutritionist Pro™ software (Axxya Systems, Stafford, TX) with the inclusion of local food items [20]; the average mean daily energy, macronutrients and micronutrients intakes were calculated. 24 h recalls were used to estimate % of energy derived from fat, carbohydrates and proteins. Underreporting was assessed using the ratio of reported energy intake to estimated basal metabolic rate (EI: BMR) (Schofield equation) [21]. A cutoff value of 0.88 was chosen based on previously used values in studies applying 24 h recalls [22].

The adoption of the Mediterranean diet was assessed with the use of the MedDietScore [23], based on information from the FFQ to reflect long-term intake. The MedDietScore assesses adherence to the Mediterranean diet based on 11 food components, each scored from 0 to 5 (non-refined cereals, fruits, vegetables, potatoes, legumes, olive oil, fish, red meat, poultry, full-fat dairy, and alcohol). The MedDietScore score ranged from 0 to 55, with higher values denoting greater adherence to the Mediterranean diet [23]. For foods aligned with the Mediterranean pattern (e.g. non-refined cereals, fruits, vegetables, fish, legumes) a score of 0 was given for rare or no consumption, 1 for 1–4 times/month, 2 for 5–8 times/month, 3 for 9–12 times/month, 4 for 13–18 times/month, and 5 for almost daily consumption. For olive oil, daily consumption received 5 points, 3–5 times/week received 4, 1–3 times/week received 3, < 1 time a week received 2, rare consumption received 1, and no consumption received 0. Foods not aligned with the Mediterranean diet (e.g., red meat, full-fat dairy) were scored on a reverse scale (i.e., 5 for no consumption). Alcohol consumption was assigned a non-linear score to reward moderation: 5 points were given for < 300 ml/day, 0 points for abstinence or high intake (> 700 ml/day), and graded scores (1–4) were assigned for intermediate intakes between 300 and 700 ml/day. The total score was then summed from all 11 components [23].

For the determination of individual FCS, an index was calculated using energy-weighted means and the following equations [24] where *i* denotes a food or beverage consumed by the participant, *FSi* the food or beverage food compass score, *Ei* the mean daily energy intake from this food or beverage, *n* the number of different foods or beverages and *fi* the daily portions of the food item or beverage.

$$ {\mathrm{FCS}} = \frac{{\mathop \sum \nolimits_{{i = 1}}^{n} FSiEi}}{{\mathop \sum \nolimits_{{i = 1}}^{n} Ei}} $$

$$\:\:Ei=fi\mathrm{*}energy\:per\:portion$$

The FCS ranges from 1 to 100 per 100 kcal with higher values indicating a better overall diet quality [6]. The FCS was calculated based on the data of the FFQ as reported in other studies [8, 15, 16].

### Assessment of the validity of the FCS

The main question addressed in the assessment of convergent construct validity was: “Does the FCS score correlate with other measures and factors that it should theoretically be related to?” If the FCS is high (meaning “healthy”), it should “converge” with high intakes of healthy nutrients (constructive components), high scores on other healthy diet indices (such as the MedDietScore), and objective biomarkers (such as erythrocyte fatty acids). In addition, according to the conceptual framework of Cronbach et al. for the assessment for construct validity three steps were followed [25] :

(i) Clearly defining the theoretical concepts and specifying the relationships among them.

Several complementary approaches were considered: associations with nutrient intakes from 24-hour recalls, associations with erythrocyte fatty acids (biomarker), and comparison with the MedDietScore. The FCS should be positively related to constructive nutrients (thiamine, riboflavin, niacin, pyridoxin, folate, cobalamin, vitamin C, vitamin A, vitamin D, Vitamin E, Vitamin K, choline, iodine, potassium, phosphorus, calcium, iron zinc, copper, magnesium, selenium, total protein, total fibre, dietary cholesterol, α-linolenic acid, eicosapentaenoic acid, docosahexaenoic acid, and total carotenoids), negatively related to sodium, and positively related to ratios included in the FCS (unsaturated: saturated fat ratio, fibre: carbohydrate ratio, potassium: sodium ratio). Moreover, the FCS should be positively related to healthy scores applying to the referenced population (mediterranean) (MedDietScore). In addition, the FCS should be positively related to gold standard biomarkers (erythrocyte omega-3 fatty acids and the omega-3 index).

(ii) Developing tools/scales to quantify the identified theoretical constructs.

Spearman partial correlations were used to examine the magnitude of associations between the continuous FCS and intakes of nutrients after energy adjustment and energy/BMR adjustment (as a measure of underreporting). Similarly, correlations of erythrocyte fatty acids and FCS were tested using Spearman coefficients. In addition, multivariable regression models were applied adjusted for age, sex and BMI.

(iii) Testing the relationships.

The pre-defined hypothesis was tested in the present sample.

### Statistical analysis

Normality was tested with the Kolmogorov-Smirnoff criterion. Normally distributed continuous variables are presented as mean values± standard deviation, while skewed variables as median and 25th -75th quartiles. Categorical variables are presented as relative frequencies (%). Non-parametric variables were log-transformed to achieve normality and do further comparisons (total body lean tissue and triacylglycerols). Variables that remained non-normally distributed after all transformations were analyzed using non-parametric methods. Comparisons between groups were performed using independent-samples t-tests for parametric or log-transformed variables, and Mann-Whitney U tests for non-parametric variables.

To assess the construct validity of the FCS, its associations with nutrients intake and fatty acids were considered. For this purpose, Spearman partial correlation coefficients adjusted for energy intake and energy intake/BMR (as measure of underreporting) as well linear regression models were applied with the FCS as dependent variable and the energy-adjusted nutrient intake or erythrocyte fatty acid as independent variable. Acceptable validity was defined as associations in the expected direction with Spearman correlation coefficients of ≥ 0.30 and < 0.50 [26]. Crude models (adjusted only for energy), age/BMI/energy adjusted models as well as age/sex/BMI/energy adjusted models were considered. Models including energy or macronutrients intake as % of energy as independent variables were not further adjusted for energy, as it had been already accounted for (not applicable). Also, models including erythrocyte fatty acids were not adjusted for energy, since they represent a biomarker and not dietary intake. The a priori selection of covariates was based on their well-established roles as major confounders in nutritional epidemiology. Of note adjusting for BMI served accounting for differences in body size (muscle mass and/or adipose tissue) that could influence nutrient intake. All reported P-values were two-sided (significance level 5%). STATA version 15 statistical software was used for the statistical analysis (STATA Corp., Texas, USA).

## Results

One hundred and six subjects (48 men and 58 women) with a mean age of 44 ± 13 years were included in the present analysis. Descriptive characteristics of the study participants are shown in Table 1. As it is shown, the mean age of the participants was 44 years, and the mean BMI was 27 kg/m^2^. The energy, macronutrient and micronutrient intake of the participants as well as the FCS and the MedDietScore are presented in Table 2. The underreporting was relatively low (*n* = 12 volunteers; 9 women and 3 men, *p* = 0.130). The main erythrocyte fatty acids are also shown in Table 3. It is noted that the omega-3 index was 5.5 ± 1.5%.

**Table 1:** Selected basic anthropometric and biochemical characteristics of the participants

	Total	Males	Females	*p*
	(*n* = 106)	(*n* = 48)	(*n* = 58)	
Age (years)	44 (13)	44 (13)	44 (14)	0.8
BMI (kg/m^2^)	27 (5)	28 (4)	26 (6)	0.2
Normal weight (%)	37.1	22.9	52.6	**0.001**
Overweight (%)	42.9	64.6	24.6	**< 0.001**
Obese (%)	17.1	10.4	22.8	0.07
Waist circumference (cm)	85.2 (14.8)	92.7 (9.2)	78.7 (15.8)	**< 0.001**
Total body fat (%)	32.6 (9.2)	26.7 (5.2)	37.2 (8.4)	**< 0.001**
Total body fat (kg)	23.5 (9.3)	21.7 (7.2)	25.1 (10.5)	0.06
Abdominal fat (DXA- Region of Interest, ROI) (kg)	2.87 (1.2)	3 (0.9)	2.7 (1.5)	0.116
Total body lean tissue (Kg)^a^	45.2 (38.6–57.1)	58.1 (52.1–64.1)	38.8 (36.2–40.6)	**< 0.001**
Total cholesterol (mmol/L)	5.5 (1.1)	5.6(0.9)	5.4 (1.1)	0.3
HDL-cholesterol (mmol/L)	1.2 (0.3)	1.1 (0.2)	1.3 (0.3)	**< 0.001**
LDL-cholesterol (mmol/L)	3.8 (0.9)	3.9 (0.7)	3.7 (0.9)	0.3
Triacylglycerols (mmol/L)^a^	0.9 (0.7–1.4)	1.3 (0.9–1.7)	0.7 (0.6-1.0)	**< 0.001**
Glucose (mmol/L)	5.1 (0.6)	5.2 (0.4)	5.0 (0.7)	**0.01**
Hemoglobin (g/dL)	14.2 (1.5)	15.2 (1.3)	13.4(1.2)	**< 0.001**
Hematocrit (%)	41.7 (4.1)	44.3 (3.5)	39.6 (3.2)	**< 0.001**
MCV (fL)	88.1 (85.4–90.6)	89.8 (85.7–91.6)	87.6 (85.2–89.9)	0.07
MCH (pg/cell)	30.3 (29.3–31.2)	30.9 (29.5–31.7)	30.0 (28.8–30.9)	**0.01**
MCHC(g/dL)	34.2 (33.5–34.7)	34.5 (33.7–34.9)	33.9 (33.4–34.4)	0.08
RDW (%)	13.7 (13.4–14.3)	13.8 (13.5–14.2)	13.6 (13.2–14.3)	0.3

Data are presented as mean ± standard deviation for normally distributed variables. Otherwise, data are presented as median (lower- upper quartile) (25th − 75th ). Student t-test or Mann Whitey test was used to compare means of normally distributed or non-normally distributed variables, correspondingly

BMI, Body mass index; MCH, mean corpuscular hemoglobin; MCHC, mean corpuscular hemoglobin concentration; RDW, Red cell distribution width

^a^ Values were log- transformed prior to statistical comparisons

Values shown in bold denote statistically significant results (*p* < 0.05)

**Table 2:** Dietary intake of participants (data from 24-h recalls)

	Total
Energy (Kcal)	1803 ± 539
*Macronutrients*	
Fat (% energy)	42.0 ± 9.2
Monounsaturated fat (% energy)	20.7 ± 7.3
Saturated fat (% energy)	12.5 ± 3.9
Polyunsaturated fat (% energy)	5.4 ± 3.9
ɑ-linolenic acid (g)	0.893 (0.6380–1.184)
Unsaturated/ saturated fatty acids ratio	2.052 (1.483–2.961)
Protein (% energy)	14.9 ± 4.1
Carbohydrate (% energy)	43.4 ± 9.9
Sugar (g)	70.6 ± 34.0
Dietary fiber (g)	17.5 ± 9.8
Dietary fiber/carbohydrate (ratio)	0.089 ± 0.0358
Dietary fiber/protein ratio	0.247 (0.172–0.354)
*Vitamins*	
Thiamin (mg)	1.2 (0.8–1.5)
Riboflavin (mg)	1.7 ± 0.7
Niacin (mg)	17.0 (9.9–22.7)
Pyridoxin (mg)	1.4 (1.0-1.8)
Pantothenic acid (mg)	3.2 (2.5–4.2)
Folate (µg)	317 (215–453)
Biotin (µg)	9.7 (3.5–18.0)
Cobalamin (µg)	3.1 (1.2–4.8)
Vitamin C (mg)	91.7 (41.5 -161.4)
Vitamin A (IU)	3436 (1918–6263)
ɑ-carotene (µg)	94.1 (14.6-274.1)
β-carotene (µg)	1426 (438–3208)
Lutein+ zeaxanthine (µg)	888 (385–2894)
Lycopene (µg)	943 (0.0-5195)
β-cryptoxanthin(µg)	98.4(4.9- 450.3)
Total carotenoids (µg)	7268 (2337–12488)
Vitamin D (IU)	50 (10–140)
Vitamin E (IU)	1.5 (0.5–2.8)
ɑ-tocopherol (mg)	7.6 (5.1–11.7)
Vitamin K (µg)	72.4 (37.3-183.8)
*Minerals*	
Iron (mg)	12.3 ± 5.4
Calcium (mg)	760 (493–1039)
Phosphorus (mg)	1041 (819–1304)
Magnesium (mg)	233 (177–311)
Manganese (mg)	1.4 (0.8–2.4)
Zinc (mg)	7.5 (5.3–10.6)
Copper (mg)	0.9 (0.7–1.3)
Chromium (mg)	0.04 (0.02–0.06)
Sodium (mg)	1639 (1027–2141)
Potassium (mg)	2396 (1673–2972)
Potassium/Sodium ratio	1.557 (0.957–2.387)
*Other*	
Cholesterol (mg)	164 (99–248)
Caffeine (mg)	64 (1.3–100)
*Quality indices*	
MedDietScore (0–55)	32.5 ± 5.2
FCS (1-100)	55.9 ± 9.5

Data are presented as mean ± standard deviation for normally distributed variables. Otherwise, data are presented as median (lower- upper quartile) (25th − 75th )

FCS, food compass score

**Table 3:** Erythrocyte content in selected fatty acids (%)

Fatty acids	% of total fatty acids
18:1n9 (oleic acid) (%)	12.7 (11.9–13.5)
20:1n9 (%)	0.26 (0.19–0.32)
24:1n9 (%)	2.99 (2.47–3.63)
20:5n3 (EPA) (%)	0.50 (0.28)
22:6n3 (DHA) (%)	5.07 (1.3)
20:4n6 (AA) (%)	12.12 (10.54–13.58)
SFA (%)	36.9 (34.9–38.0)
MUFA (%)	17.89 (3.2)
PUFA (%)	35.2 (2.4)
n-6 (%)	27.3 (3.1)
n-3 (%)	7.7 (1.7)
Omega-3 index	5.5 (1.5)

Data are presented as mean ± standard deviation for normally distributed variables. Otherwise, data are presented as median (lower- upper quartile) (25th − 75th )

AA, arachidonic acid; DHA, docosahexaenoic acids; EPA, eicosapentaenoic acid; MUFA, monounsaturated fatty acids; PUFA, polyunsaturated fatty acids; SFA, saturated fatty acids

The FCS was positively related to age of the participants (Pearson rho = 0.393, p = < 0.001), while no relation was found between FCS and energy intake (Pearson rho = − 0.103, *p* = 0.3) or BMI (Pearson rho =-0.068, *p* = 0.5). Supplementary Tables 1 and 2 present Spearman partial correlation coefficients between FCS and nutrients/ MedDietScore adjusted for energy and energy/BMR, respectively. In energy adjusted models the FCS was significantly associated to MUFA (% energy) (rho = 0.247, *p* = 0.02), unsaturated/ SFA ratio (rho = 0.307, *p* = 0.004), dietary fiber/carbohydrate (ratio) (rho = 0.380, *p* < 0.001), potassium/sodium ratio (rho = 0.260, *p* = 0.02), cholesterol (mg) (rho= -0.256, *p* = 0.018), vitamin C (mg) (rho = 0.271, *p* = 0.01), vitamin A (IU) (rho = 0.244, *p* = 0.02), β-carotene (µg) (rho = 0.276, *p* = 0.01), Lutein+ zeaxanthine (µg) (rho = 0.326, *p* = 0.002), ɑ-tocopherol (mg) (rho = 0.302, *p* = 0.009), Vitamin K (µg) (rho = 0.326 *p* = 0.005), and MedDietScore (rho = 0.386, *p* < 0.001). Adjustment for energy/BMR yielded similar results. Acceptable validity was defined as associations in the expected direction with Spearman correlation coefficients of ≥ 0.30 and < 0.5 [26]. The correlation coefficients of the following nutrients with the FCS were ≥ 0.3: Unsaturated/ SFA ratio, Dietary fiber/carbohydrate ratio, Lutein+ zeaxanthine (µg), ɑ-tocopherol (mg), and Vitamin K (µg). A strong positive correlation was observed between the FCS and the MedDietScore (rho = 0.386, *p* < 0.001), indicating that both indices capture similar aspects of dietary quality. It is noted that all correlations were in the expected direction. In Table 4 crude, age-BMI and age-sex-BMI adjusted regression coefficients of the FCS and nutrient intake from 24 h recalls are displayed. In the total sample, the FCS was positively associated with % total fat intake, % MUFA, vitamin C, α-tocopherol, vitamin K and potassium intakes, in fully adjusted models. The FCS was negatively associated with % carbohydrate intake. In addition, the FCS was positively related to MedDietScore.

**Table 4:** Crude (energy adjusted), age-BMI- energy, and age-sex-BMI-energy adjusted regression coefficients of the FCS and dietary parameters

	Crude coefficients energy adjusted				Age-BMI -energy adjusted coefficients				Age-sex-BMI-energy adjusted coefficients			
	β	95% CI	Stand β	*p*	β	95% CI	Stand β	*p*	β	95% CI	Stand β	*p*
Energy (Kcal) ╪	− 0.002	− 0.006 0.002	− 0.103	0.3	0.001	− 0.002 0.005	− 0.254	0.457	0.003	− 0.001 0.007	0.183	0.1
*Macronutrients*												
Fat (% energy) ╪	0.196	− 0.020 0.412	0.193	0.07	0.253	0.059 0.447	0.252	**0.01**	0.230	0.036 0.425	0.229	**0.02**
MUFA (% energy) ╪	0.448	0.190 0.706	0.353	**0.001**	0.419	0.186 0.652	0.333	**0.001**	0.401	0.170 0.632	0.319	**0.001**
SFA (% energy) ╪	− 0.436	− 0.951 0.078	− 0.181	0.09	− 0.053	− 0.574 0.468	− 0.022	0.8	− 0.125	− 0.642 0.392	− 0.052	0.6
PUFA (% energy) ╪	0.448	− 0.564 10.460	0.096	0.3	0.148	− 0.802 1.099	0.032	0.7	0.082	− 0.856 1.020	0.018	0.8
ɑ-linolenic acid (g)	3.348	− 1.593 8.288	0.217	0.1	1.377	− 3.287 6.041	0.090	0.5	0.230	− 4.417 4.876	0.015	0.9
Unsaturated/ SFA ratio∫	3.036	1.185 4.887	0.335	**0.02**	2.133	0.293 3.972	0.238	**0.024**	2.180	0.400 3.960	0.243	**0.017**
Protein (% energy)	− 0.135	− 0.619 0.349	− 0.060	0.5	0.219	− 0.250 0.688	0.099	0.3	0.182	− 0.282 0.646	0.082	0.4
Carbohydrate (% energy)	− 0.099	− 0.300 0.101	− 0.107	0.3	− 0.226	− 0.409 − 0.044	− 0.245	**0.01**	− 0.209	− 0.391 − 0.027	− 0.227	**0.025**
Total sugars (g)	0.011	− 0.065 0.086	0.039	0.7	− 0.012	− 0.082 0.058	− 0.045	0.7	0.008	− 0.062 0.078	0.029	0.8
Dietary fiber (g)	0.277	0.04 0.508	0.294	**0.01**	0.114	− 0.118 0.346	0.122	0.3	0.146	− 0.080 0.371	0.156	0.2
Dietary fiber/carbohydrate (ratio)	95.558	43.322 147.793	0.369	**< 0.001**	69.430	18.367 120.492	0.271	**0.008**	68.551	19.048 118.054	0.267	**0.007**
Dietary fiber/protein ratio	9.790	− 1.139 20.719	0.191	0.07	0.776	− 10.286 11.838	0.015	0.8	3.512	− 7.430 14.454	0.069	0.5
*Vitamins*												
Thiamin (mg)	− 4.484	− 8.782 − 0.185	− 0.322	**0.04**	− 3.473	− 7.469 0.523	− 0.252	0.08	− 3.330	− 7.214 0.555	− 0.241	0.09
Riboflavin (mg)	− 1.128	− 4.55 2.30	− 0.090	0.5	− 0.591	− 3.928 2.746	− 0.046	0.7	− 1.081	− 4.341 2.178	− 0.085	0.5
Niacin (mg)	− 0.108	− 0.301 0.085	− 0.145	0.2	− 0.034	− 0.224 0.156	− 0.045	0.7	− 0.040	− 0.224 0.145	− 0.053	0.6
Pyridoxin (mg)	− 0.276	− 2.839 2.287	− 0.030	0.8	− 0.303	− 2.656 2.049	− 0.033	0.798	− 0.736	− 3.043 1.571	− 0.081	0.5
Pantothenic acid (mg)	0.415	− 0.844 1.674	0.084	0.5	0.083	− 1.113 1.279	0.017	0.8	− 0.150	− 1.328 1.027	− 0.030	0.2
Folate (µg)	0.004	− 0.006 0.014	0.093	0.4	− 0.00002	− 0.009 0.009	− 0.001	0.9	0.001	− 0.008 0.010	0.023	0.8
Biotin (µg)	0.088	− 0.082 0.258	0.119	0.6	0.036	− 0.121 0.194	0.050	0.6	0.040	− 0.114 0.193	0.054	0.6
Cobalamin (µg)	0.246	− 0.055 0.547	0.177	0.1	0.219	− 0.107 0.545	0.139	0.185	0.166	− 0.155 0.487	0.105	0.3
Vitamin C (mg)	0.027	0.008 0.046	0.316	**0.005**	0.019	0.001 0.037	0.009	**0.03**	0.022	0.005 0.038	0.255	**0.01**
Vitamin A (IU)	0.00007	− 0.00013 0.00022	0.095	0.3	0.00003	− 0.00013 0.00020	0.026	0.672	0.00006	− 0.00011 0.00023	0.073	0.4
β-carotene (µg)	0.00012	− 0.0001 0.0004	0.081	0.3	0.00002	− 0.0002 0.0003	0.017	0.8	0.00008	− 0.00020 0.00038	0.063	0.5
Lutein+ zeaxanthine (µg)	0.00004	0.0001 0.0004	0.051	0.4	− 0.000004	− 0.00017 0.00016	− 0.005	0.9	0.00002	− 0.00014 0.0019	0.030	0.7
Lycopene (µg)	0.00005	− 0.0004 0.0005	0.026	0.8	0.00004	− 0.0003 0.0004	0.022	0.8	0.00007	− 0.00034 0.0004	0.035	0.7
β-cryptoxanthin (µg)	0.002	− 0.002 0.006	0.115	0.2	0.0003	− 0.003 0.004	0.020	0.8	0.002	− 0.002 0.006	0.091	0.3
Total caroteinoids (µg)	0.000047	− 0.00006 0.00016	0.095	0.3	0.000003	− 0.000099 0.000106	0.008	0.9	0.000011	− 0.00009 0.00011	0.024	0.8
Vitamin D (IU)	− 0.005	− 0.032 0.022	− 0.045	0.7	− 0.002	− 0.029 0.025	− 0.015	0.8	0.005	− 0.020 0.031	0.048	0.6
Vitamin E (IU)	− 0.359	− 1.604 0.885	− 0.065	0.5	− 0.346	− 1.370 0.678	− 0.073	0.5	0.210	− 0.957 1.377	0.038	0.7
ɑ-tocopherol (mg)	0.705	0.281 1.129	0.385	**0.001**	0.387	− 0.054 0.828	0.214	0.08	0.427	0.070 0.785	0.236	**0.02**
Vitamin K (µg)	4.930	1.053 8.806	0.313	**0.04**	5.255	1.670 8.840	0.280	**0.005**	5.255	1.670 8.840	0.062	**0.005**
*Minerals*												
Iron (mg)	− 0.060	− 0.565 0.444	− 0.035	0.8	− 0.099	− 0.560 0.361	0.058	0.6	0.119	− 0.241 0.478	0.069	0.5
Calcium (mg)	0.00009	− 0.006 0.005	− 0.004	0.9	0.001	− 0.004 0.006	0.052	0.8	0.001	− 0.004 0.006	0.056	0.5
Phosphorus (mg)	0.001	− 0.005 0.008	0.070	0.6	0.002	− 0.004 0.008	0.075	0.6	0.003	− 0.002 0.007	0.121	0.2
Magnesium (mg)	0.008	− 0.012 0.027	0.105	0.4	0.004	− 0.011 0.019	0.050	0.9	0.008	− 0.007 0.023	0.110	0.2
Manganese (mg)	0.929	− 0.594 2.453	0.140	0.2	0.083	− 1.381 1.547	0.013	0.9	0.205	− 1.220 1.631	0.031	0.7
Zinc (mg)	− 0.513	− 0.323 0.297	− 0.211	0.2	− 0.008	− 0.791 0.774	− 0.003	0.9	0.354	− 0.209 0.918	0.147	0.3
Copper (mg)	1.532	− 0.105 3.169	0.206	0.06	0.928	− 0.883 2.740	0.110	0.3	0.854	− 0.908 2.616	0.101	0.3
Chromium (mg)	1.532	− 48.272 60.367	0.128	0.8	− 5.183	− 55.327 44.962	− 0.024	0.8	2.239	− 2.665 7.142	0.638	0.3
Sodium (mg)	− 0.001	− 0.004 0.001	− 0.139	0.3	− 0.001	− 0.003 0.002	− 0.054	0.6	0.001	− 0.002 0.003	0.051	0.6
Potassium (mg)	0.003	0.001 0.006	0.361	**0.012**	0.002	− 0.001 0.004	0.198	0.1	0.002	0.0008 0.004	0.209	**0.04**
Potassium/Sodium ratio	2.220	0.705 3.734	0.304	**0.005**	1.816	0.409 3.223	0.251	**0.012**	1.834	0.473 3.196	0.253	**0.009**
*Other*												
Cholesterol (mg)	− 0.010	− 0.024 0.005	− 0.158	0.19	− 0.004	− 0.017 0.010	− 0.061	0.5	− 0.002	− 0.016 0.011	− 0.041	0.7
Caffeine (mg)	− 0.020	− 0.038 − 0.003	− 0.238	**0.02**	− 0.017	− 0.035 0.001	− 0.198	0.05	− 0.016	− 0.033 0.001	− 0.185	0.07
*Diet quality scores*												
MedDietScore	0.990	0.669 1.310	0.538	**< 0.001**	0.830	0.498 1.162	0.455	**< 0.001**	0.805	0.473 1.137	0.441	**< 0.001**

Energy adjustment was applied in all models (except for energy and macronutrients expressed as % of energy

FCS, food compass score; MUFA, monounsaturated fatty acids; PUFA, polyunsaturated fatty acids; SFA, saturated fatty acids

∫ Unsaturated fat represent the sum of monounsaturated and polyunsaturated fatty acids

╪ Not adjusted for energy

Values shown in bold denote statistically significant results (*p* < 0.05)

Supplementary Table 3 presents Spearman correlation coefficients between FCS and erythrocyte fatty acids. It is noted that the FCS was correlated with 18:1n9 (oleic acid) (rho= − 0.207, *p* = 0.04), 24:1n9 (rho = 0.217, *p* = 0.03), 20:5n3 (EPA) (rho = 0.411, *p* < 0.001), 22:6n3 (DHA) (rho = 0.343, *p* = 0.001), n-6 (rho = 0.368, *p* < 0.001), and the omega-3 index (rho = 0.363, *p* < 0.001). The Spearman correlation coefficients of the following erythrocyte fatty acids with the FCS were ≥ 0.3 (accepted validity): 20:5n3 (EPA), 22:6n3 (DHA), n-6, and Omega-3 index. It is noted that all correlations were in the expected direction. In Table 5 age-BMI as well as age-sex-BMI adjusted regression coefficients of the FCS and erythrocyte fatty acids are shown. Ιn models adjusted for age, sex and BMI, the FCS was positively associated with the % content of erythrocytes in EPA (B = 13.814, *p* = 0.001), DHA (B = 1.769, *p* = 0.01), and omega-3 fatty acids (B = 1.380, *p* = 0.01). Similarly, the omega-3 index was positively related to the FCS (B = 1.581, *p* = 0.01).

**Table 5:** Crude, age-BMI and age, sex, BMI adjusted regression coefficients of the FCS and selected erythrocyte fatty acids

% of total fatty acids	Crude coefficients				Age and BMI adjusted coefficients				Age, sex and BMI adjusted coefficients			
	β	95% CI	Stand β	*p*	β	95% CI	Stand β	*p*	β	95% CI	Stand β	*p*
18:1n9 (oleic acid)	− 0.143	− 0.811 0.525	− 0.044	0.6	− 0.286	− 0.900 0.329	− 0.089	0.3	− 0.196	− 0.812 0.421	− 0.061	0.5
20:1n9	3.115	− 16.07 22.30	0.033	0.7	− 7.171	− 25.58 11.24	− 0.077	0.4	− 6.447	− 24.65 11.75	− 0.069	0.4
24:1n9	1.924	− 0.208 4.056	0.183	0.07	0.943	− 1.134 3.020	0.089	0.3	0.968	− 1.081 3.018	0.092	0.3
20:4n6	0.199	− 0.915 1.313	0.037	0.7	1.157	0.072 2.241	0.213	**0.03**	1.024	− 0.065 2.114	0.189	0.06
20:5n3 (EPA)	18.163	10.520 25.806	0.399	**0.00001**	13.03	4.708 21.371	0.259	**0.003**	13.814	5.632 21.996	0.274	**0.001**
22:6n3 (DHA)	2.676	1.288 4.063	0.369	**0.0002**	1.669	0.210 3.127	0.232	**0.02**	1.769	0.332 3.205	0.246	**0.01**
SFA	0.524	− 0.223 1.272	0.143	0.1	0.437	− 0.251 1.126	0.120	0.2	0.467	− 0.212 1.147	0.128	0.1
MUFA	0.047	− 0.549 0.642	0.016	0.8	− 0.264	− 0.833 0.306	− 0.092	0.3	− 0.194	− 0.763 0.375	− 0.068	0.5
PUFA	0.196	− 0.598 0.990	0.051	0.6	0.744	− 0.017 1.506	0.193	**0.05**	0.723	− 0.030 1.475	0.188	0.06
n-6	− 0.518	− 1.149 0.114	− 0.167	0.1	0.123	− 0.545 0.790	0.040	0.7	0.077	− 0.584 0.738	0.025	0.8
n-3	2.007	0.986 3.027	0.375	**0.0001**	1.289	0.225 2.353	0.243	**0.01**	1.380	0.333 2.428	0.260	**0.01**
Omega-3 index	2.354	1.187 3.521	0.384	**0.0001**	1.492	0.249 2.735	0.245	**0.01**	1.581	0.358 2.805	0.259	**0.01**

FCS, food compass score

DHA, docosahexaenoic acid; EPA, eicosapentaenoic acid; MUFA, monounsaturated fatty acids; PUFA, polyunsaturated fatty acids; SFA, saturated fatty acids

Omega-3 index: Sum of EPA + DHA

Values shown in bold denote statistically significant results (*p* < 0.05)

## Discussion

The present study is the first one to validate the newly developed FCS with dietary intake and nutritional biomarkers. The FCS demonstrated good convergent construct validity, as evidenced by its significant associations (Spearman rho ≥ 0.3) in the hypothesized directions with key nutrients (unsaturated/ SFA ratio, dietary fiber/carbohydrate ratio, lutein+ zeaxanthin, ɑ-tocopherol, and Vitamin K), MedDietScore, and objective biomarkers (EPA, DHA, n-6, and Omega-3 index). In addition, the FCS was associated with dietary fat intake, unsaturated/ SFA ratio, dietary fiber/carbohydrate ratio, α-tocopherol, vitamin K, vitamin C, potassium, potassium/sodium ratio, and erythrocyte omega-3 after adjustment for age, sex, BMI and energy intake (where applicable).

The FCS in the present study was higher than that reported for university students (median 56 vs. 47.6) [8] and similar to apparently healthy Greek adults [27]. The MedDietScore of the present sample was also higher than that previously reported for Greek university students (median 32 vs. 30) [8]. Although different FFQs were used in these studies and the time-periods considered were almost one decade apart, our results indicate a better dietary quality in this sample compared to university students. The FCS was positively associated with the MedDietScore, which has been previously reported by our group [8]. It is also noted that the observed correlation coefficient of FCS and MedDietScore (rho > 0.9) was higher than that reported for FCS and Nutri-Score or Health Star Rating (rho = 0.66 and 0.7. respectively) [6]. Similarly, the FCS-MedDietScore correlation coefficient was higher than that reported between Nutri-Score, the Canadian ‘High In’ Symbol, and the Diabetes Canada Clinical Practice Guidelines (DCCP) (rho ~ 0.6–0.7) [28]. The Nutri-Score was even negatively related to Mediterranean diet adherence in an Italian cohort [29]. The close “bond” of the FCS to the MedDietScore along with the evidence-based benefits of the Mediterranean diet [30], favor its potential use in Μediterranean populations. In parallel, national authorities in several Mediterranean countries have raised concerns regarding the compatibility of the Nutri-Score with traditional Mediterranean dietary patterns [31].

In general, NPS-based indices- while developed for specific purposes- have been extensively validated against clinical outcomes, such as cancer, cardiovascular disease, and mortality [32]. According to a recent meta-analysis the Nutri-Score had substantial criterion validation evidence, while FCS and other NPS-based indices had intermediate criterion validation evidence so far [13]. Indeed, FCS has been inversely related to inflammatory indices [27] and clinical outcomes, such as obesity [16], dyslipidemia [16], cardiovascular disease [17] and mortality [17]. It has been suggested that further validation studies in diverse contexts should be conducted [13].

Notably, the relationship of NPS scores with dietary intake and nutritional biomarkers is less studied. Higher Food Standards Agency modified NPS (FSAmNPS) index (used for Nutri-Score calculation) have been related to lower MUFA-to-SFA fat ratio, MUFA, fiber, protein and higher energy, total fat, saturated fat, polyunsaturated fat, and dietary cholesterol intake [29, 33, 34]. Previous studies have mostly assessed the association of the FCS with validated healthy patterns, such as the Healthy Eating Index [17] and the MedDietScore [8], as well as food-groups intake [6, 8], showcasing its ability to capture the healthiness of diet as a whole. In these studies FCS was calculated from 24 h recalls [17] or an FFQ [8].

In the present study, the FCS was positively related to % fat and MUFA intake. Moreover, erythrocyte EPA and DHA, as well as the omega-3 index were positively associated with the score. As mature erythrocytes have not the ability for *de novo* lipid synthesis, their phospholipid content reflects circulating lipoproteins [35]. Interestingly, their carrying fatty acids may be related to several pathophysiological states [35]. Our observations are in line with the design methodology of the FCS and support its concept [6]. Indeed, the FCS incorporates the unsaturated: SFA ratio as well as individual fatty acids (ɑ-linolenic acid EPA, DHA and medium-chain fatty acids) [6]. In addition, the food groups considered for the FCS such as nuts and seeds, seafood and plant oils are rich in MUFA or PUFA [20], while the FCS has been previously associated with olive oil consumption [8]. On the contrary, higher FSAm-NPS values (reflecting lower diet quality) have been related to lower dietary intake of MUFA and higher dietary intake of PUFA [29, 33, 34], underlying the different constructive concepts of FCS and Nutri-Score regarding fat. However, no relation was found between the FCS and erythrocytes MUFA or SFA content. Although erythrocytes PUFA reflect the PUFA status of the human body constituting a valid biomarker [36, 37], their MUFA and SFA content are not so well correlated with their intake from the diet since they can be also *de novo* produced [38]. For example, oleic acid in the body can also be synthesized from stearic acid [39] and under conditions of essential fatty acids deficiency, it can be elongated to mead acid (20:3n9) [40]. The negative association between the FCS and carbohydrates may derive from the fact that only dietary fiber and not total carbohydrates are positively scored [6]. Moreover, the consumption of processed carbohydrate-based foods, high in preservatives would be also a negative attribute to the score [6]. Indeed, the FCS has been previously reported to be negatively related to refined grains consumption [8].

For the design of the FCS the following vitamins have been taken into account: Vitamin A, thiamin, riboflavin, niacin, pyridoxin, folate, cobalamin, vitamin C, vitamin D, vitamin E, vitamin K and choline [6]. In addition, nutrients ratios such as unsaturated/ SFA ratio, dietary fiber/carbohydrate ratio, dietary fiber/protein ratio, and potassium/sodium ratio are included in the algorithm [6]. In the present study, positive associations of the FCS and nutrients (vitamins C, ɑ-tocopherol and potassium) as well as nutrient ratios (unsaturated/ SFA ratio, dietary fiber/carbohydrate, potassium/sodium ratio) were documented. Similar associations have been documented at the validating process of other quality scores [41, 42]. Interestingly, with the exception of vitamin C, the FCS is mostly associated with lipid-soluble vitamins. This may be related to its ability to reflect dietary intake and circulating lipids, as discussed above.

Modest associations with minerals were found in the present study, although they are included in the FCS scoring. In other studies validating dietary scores, no association was found with iron and total folate [43] or negative associations have been documented between scores and iron, zinc, cobalamin, cholesterol and SFA [42], which possibly reflects the negative scoring of meat in scores as in the case of the FCS [6]. It is noted that a negative association of the FCS with red meat intake has been previously reported [8]. Interestingly, in scores where threshold for meat consumption is applied (i.e. increasing points for meat until specific consumed amounts, after which no points are given), positive associations with iron, zinc and cobalamin have been reported [41].

A wide range in the magnitude of regression coefficients was observed, in line with other studies [41, 43], which is explained through differences in unit measurements and/or by the fact that the FCS is composed by many variables and each one is expected to have a low impact on the total score [6]. In addition, significant associations (Spearman rho ≥ 0.3) were identified for several constructive nutrients and ratios.

The strengths of our study include the validation of a brand-new index, the FCS score, which uses expanded evidence-based characteristics for assessing diet quality [6]. The FCS considers several food characteristics and scores 54 different items uniformly using the same algorithm and cut-off points [6]. In addition, energy, BMI and sex-adjusted correlations are presented, while most studies investigating the relationships of NPS to dietary intakes report simple correlation coefficients, with some exceptions [29]. This difference is important since associations with macro- and micro-nutrient intakes may be mediated through higher energy intakes. Several studies with NPS (such as those regarding Nutri-Score), have been mostly carried out by their developers [44]. However, in the present study, the authors were not related to the developers of the FCS. Of note, all dietary information was collected through interviews with dietitians, which increases data quality [26]. Indeed, according to the criteria of Serra-Majem et al. for the evaluation of the quality of dietary intake validation studies, the present study scores adequately (acceptable quality) [26]. In addition, subjects were not taking supplements, so the association of erythrocyte omega-3 fatty acids purely reflects dietary intake. This makes the biomarker particularly appropriate for validating nutrient intake and related indices, such as the FCS.

Several limitations should be considered. Firstly, the cross-sectional design of our study cannot support causality. Moreover, several errors may have arisen during dietary assessment. The healthy volunteer effect is possible, meaning that participants may have been healthier or more health-conscious than the general population, potentially biasing dietary intake patterns and FCS calculations. Moreover, the FFQ was administered only once and subjects may not have appropriately reported their food consumption, which may be related to altered FCS calculations. However, we have partially accounted for this error by inserting energy intake as a confounding variable in the reported correlations, as methodologically suggested for validation studies [26]. Moreover, the FFQ includes standard items, and it may not include particular foods, which are commonly consumed by the participants. For the validation of the FCS, however, multiple 24 h recalls were used, which better capture dietary intake. Seasonal variability in dietary intake was not accounted for, which could have influenced the observed nutrient patterns and FCS calculations. In addition, no correction for multiple testing was applied. Although the analyses were based on predefined set of dietary variables, the possibility of Type I error cannot be excluded .Finally, the FCS estimation is based on US foods and differences may exist concerning Greek traditional foods and the sample size is relatively small. It is also noted that the FCS also contains other aspects of diet (such as additives intake), which could not be captured through the present study design.

## Conclusion

The present study represents the first one to nutritionally validate the FCS since its proposal in 2021. The investigated correlations with nutrients and erythrocyte fatty acids suggest that FCS is a valid index with an additive value for clinical use and/or consumer guidance. Future studies assessing the FCS against health outcomes could further enhance its use as a tool to formulate food policy and progress to new product development. 

## Supplementary Information

Below is the link to the electronic supplementary material.

Supplementary Material 1

## Data Availability

Data are available from the corresponding author upon reasonable request.

## Abbreviations

AA: Arachidonic acid
ΒΜΙ: Body mass index
BMR: Basal metabolic rate
DHA: Docosahexaenoic acid
DXA: Dual- energy-X-ray absorptiometry
EPA: Eicosapentaenoic acid
FAME: Fatty acids methyl esters
FCS: Food compass score
FFQ: Food frequency questionnaire
FSA: Food standards agency
MCH: Mean corpuscular hemoglobin
MCHC: Mean corpuscular hemoglobin concentration
MET: Metabolic equivalents
MUFA: Monounsaturated fatty acids
NPS: Nutrient profiling systems
PUFA: Polyunsaturated fatty acids
RBC: Red blood cells
RDW: Red cell distribution width
ROI: Region of interest
SFA: Saturated fatty acids
