# Supplementary material for: Validation of the food compass score through 24 h recalls and measurement of erythrocyte fatty acids in a mediterranean population
Source: Eur J Nutr. 2026 Feb 25;65(2):68. doi: 10.1007/s00394-026-03912-0 (PMC12935804; doi:10.1007/s00394-026-03912-0)
Supplement: Supplementary file 1 — Supplementary Material 1 [file 394_2026_3912_MOESM1_ESM.doc]

**Supplementary Table 1: Spearman partial correlation coefficients between FCS macro- and micro- nutrients (adjusted for energy).**

|  | **Spearman rho** | | **p-value** |  |  | **Spearman rho** | **p-value** |
| --- | --- | --- | --- | --- | --- | --- | --- |
| ***Macronutrients*** |  | |  |  | ***Minerals*** |  |  |
| Fat ╪  (% energy) | 0.114 | | 0.2 |  | Iron (mg) | -0.011 | 0.9 |
| MUFA ╪  (% energy) | **0.247** | | **0.02** |  | Calcium (mg) | -0.028 | 0.8 |
| SFA ╪  (% energy) | -0.198 | | 0.06 |  | Phosphorus (mg) | 0.064 | 0.5 |
| PUFA ╪  (% energy) | 0.095 | | 0.3 |  | Magnesium (mg) | 0.109 | 0.3 |
| ɑ-linolenic acid (g) | 0.031 | | 0.7 |  | Manganese (mg) a | 0.153 | 0.1 |
| Unsaturated/ SFA ratio∫ | **0.307** | | **0.004** |  | Zinc (mg) | -0.001 | 0.9 |
| Protein ╪  (% energy) | -0.06 | | 0.5 |  | Copper (mg) a | 0.165 | 0.1 |
| Carbohydrate╪ (% energy) | -0.051 | | 0.6 |  | Chromium (mg)a | 0.081 | 0.4 |
| Sugar (g) | -0.070 | | 0.5 |  | Sodium (mg) | -0.200 | 0.07 |
| Dietary fiber (g) | 0.169 | | 0.1 |  | Potassium (mg) | 0.193 | 0.08 |
| Dietary fiber/carbohydrate (ratio) | **0.380** | | **<0.001** |  | Potassium/Sodium ratio | **0.260** | **0.02** |
| Dietary fiber/protein ratio | 0.202 | | 0.06 |  |  |  |  |
| ***Vitamins*** |  |  | |  | ***Other*** |  |  |
| Thiamin (mg) | -0.177 | 0.1 | |  | Cholesterol (mg) a | **-0.256** | **0.018** |
| Riboflavin (mg) | -0.115 | | 0.2 |  | Caffeine (mg) | -0.182 | 0.09 |
| Niacin (mg) | -0.109 | | 0.3 |  |  |  |  |
| Pyridoxin (mg) | 0.03 | | 0.7 |  |  |  |  |
| Pantothenic acid (mg) | 0.15 | | 0.1 |  | MedDietScore | **0.386** | **<0.001** |
| Folate (ug) | 0.103 | | 0.3 |  |  |  |  |
| Biotin (ug) | 0.119 | | 0.2 |  |  |  |  |
| Covalamin (ug) | 0.042 | | 0.7 |  |  |  |  |
| Vitamin C (mg) | **0.271** | | **0.01** |  |  |  |  |
| Vitamin A (IU) | **0.244** | | **0.02** |  |  |  |  |
| β-carotene (ug) | **0.276** | | **0.01** |  |  |  |  |
| Lutein+ zeaxanthine (ug) | **0.326** | | **0.002** |  |  |  |  |
| Lycopene (ug) | 0.203 | | 0.06 |  |  |  |  |
| β-cryptoxanthin (ug) | 0.062 | | 0.5 |  | - |  |  |
| Total caroteinoids (ug) | 0.194 | | 0.07 |  |  |  |  |
| Vitamin D (IU) | -0.114 | | 0.3 |  |  |  |  |
| Vitamin E (IU) | -0.107 | | 0.3 |  |  |  |  |
| ɑ-tocopherol (mg) | **0.302** | | **0.009** |  |  |  |  |
| Vitamin K (ug) | **0.326** | | **0.005** |  |  |  |  |

FCS: Food Compass Score; MUFA: monounsaturated fatty acids; PUFA: polyunsaturated fatty acids; SFA: saturated fatty acids.

∫ Unsaturated fat represent the sum of monounsaturated and polyunsaturated fatty acids

╪ No energy adjustment as already included in the variable.

**Supplementary Table 2: Spearman partial correlation coefficients between FCS, macro- and micro- nutrients (adjusted for energy/BMR as a measure of underreporting).**

|  | **Spearman rho** | | **p-value** |  |  | **Spearman rho** | **p-value** |
| --- | --- | --- | --- | --- | --- | --- | --- |
| ***Macronutrients*** |  | |  |  | ***Minerals*** |  |  |
| Fat ╪  (% energy) | 0.114 | | 0.2 |  | Iron (mg) | -0.041 | 0.7 |
| MUFA ╪  (% energy) | **0.247** | | **0.02** |  | Calcium (mg) | -0.036 | 0.7 |
| SFA ╪  (% energy) | -0.198 | | 0.06 |  | Phosphorus (mg) | 0.032 | 0.7 |
| PUFA ╪  (% energy) | 0.095 | | 0.3 |  | Magnesium (mg) | 0.074 | 0.5 |
| ɑ-linolenic acid (g) | 0.012 | | 0.913 |  | Manganese (mg) a | 0.132 | 0.2 |
| Unsaturated/ SFA ratio∫ | **0.306** | | **0.005** |  | Zinc (mg) | -0.031 | 0.7 |
| Protein ╪  (% energy) | -0.06 | | 0.5 |  | Copper (mg) a | 0.135 | 0.2 |
| Carbohydrate ╪ (% energy) | -0.051 | | 0.6 |  | Chromium (mg)a | 0.062 | 0.5 |
| Sugar (g) | -0.084 | | 0.449 |  | Sodium (mg) | **-0.221** | **0.05** |
| Dietary fiber (g) | 0.157 | | 0.154 |  | Potassium (mg) | 0.164 | 0.1 |
| Dietary fiber/carbohydrate (ratio) | **0.383** | | **<0.001** |  | Potassium/Sodium ratio | **0.266** | **0.01** |
| Dietary fiber/protein ratio | 0.205 | | 0.06 |  |  |  |  |
| ***Vitamins*** |  |  | |  | ***Other*** |  |  |
| Thiamin (mg) | -0.189 | 0.08 | |  | Cholesterol (mg) a | **-0.270** | **0.01** |
| Riboflavin (mg) | -0.146 | | 0.1 |  | Caffeine (mg) | -0.201 | 0.06 |
| Niacin (mg) | -0.133 | | 0.2 |  |  |  |  |
| Pyridoxin (mg) | 0.003 | | 0.9 |  |  |  |  |
| Pantothenic acid (mg) | 0.125 | | 0.2 |  | MedDietScore | **0.391** | **<0.001** |
| Folate (ug) | 0.084 | | 0.4 |  |  |  |  |
| Biotin (ug) | 0.104 | | 0.3 |  |  |  |  |
| Covalamin (ug) | 0.020 | | 0.8 |  |  |  |  |
| Vitamin C (mg) | **0.256** | | **0.01** |  |  |  |  |
| Vitamin A (IU) | **0.240** | | **0.02** |  |  |  |  |
| β-carotene (ug) | **0.273** | | **0.01** |  |  |  |  |
| Lutein+ zeaxanthine (ug) | **0.320** | | **0.003** |  |  |  |  |
| Lycopene (ug) | 0.196 | | 0.07 |  |  |  |  |
| β-cryptoxanthin (ug) | 0.052 | | 0.6 |  | - |  |  |
| Total caroteinoids (ug) | 0.187 | | 0.08 |  |  |  |  |
| Vitamin D (IU) | -0.118 | | 0.321 |  |  |  |  |
| Vitamin E (IU) | -0.103 | | 0.385 |  |  |  |  |
| ɑ-tocopherol (mg) | **0.278** | | **0.01** |  |  |  |  |
| Vitamin K (ug) | **0.324** | | **0.005** |  |  |  |  |

FCS: Food Compass Score; MUFA: monounsaturated fatty acids; PUFA: polyunsaturated fatty acids; SFA: saturated fatty acids.

∫ Unsaturated fat represent the sum of monounsaturated and polyunsaturated fatty acids.

╪ No energy adjustment as already included in the variable.

**Supplementary Table 3: Spearman correlation coefficients between FCS and erythrocyte fatty acids.**

|  |  |  |
| --- | --- | --- |
| **% of total fatty acids** | **Spearman rho** | **P-value** |
| **18:1n9 (oleic acid)** | **-0.207** | **0.04** |
| **20:1n9** | 0.142 | 0.1 |
| **24:1n9** | **0.217** | **0.03** |
| **20:4n6** | 0.034 | 0.7 |
| **20:5n3 (EPA)** | **0.411** | **<0.001** |
| **22:6n3 (DHA)** | **0.343** | **0.001** |
| **SFA** | 0.094 | 0.3 |
| **MUFA** | 0.133 | 0.1 |
| **PUFA** | 0.042 | 0.6 |
| **n-6** | **0.368** | **<0.001** |
| **n-3** | -0.166 | 0.1 |
| **Omega-3 index** | **0.363** | **<0.001** |

FCS: Food Compass Score
